# Extra Supplement—The Nursing Mirror

**Published:** 1889-07-06

**Authors:** 


*The Hospital*, July 6, 1889.

### The Nursing Mirror.

[*Extra Supplement*

All Contributions for this Supplement should be addressed "The Nursing Mirror," care of the Editor, 139-140, Salisbury Court, Fleet Street, London, E.C.

### En Passant.

IN SEASON AND OUT OF SEASON.—The last quarterly letter to the Mary Adelaide nurses is written by Miss Wood, who seizes the opportunity of advertising her pet association, and dragging it in by the heels on almost every page. This is a pity, for the letter is well-written and full of the most commendable sentiments. During the last six months twenty-six of the Mary Adelaide nurses have received appointments.

REGISTRATION.—The promoters of the present scheme of registration for nurses must be feeling rather uncomfortable at present, under the shower of criticism poured upon their unfortunate endeavours. Dr. Sansom's letter, printed in the *British Medical Journal*, and which we produce elsewhere, is a specimen of the discredit into which the British Nurses' Association has fallen. Doctors were most ready to join any sensible scheme for the benefit of nurses, and willingly lent their names to an association which they thought would work towards that end; but now that plans are promulgated which authorities declare to be injurious to the best interests of nurses, the doctors naturally withdraw their names. In the *Pall Mall* there was an article by a physician in which he pointed out that "as for the nurses themselves, at present they pay nothing a year for certificates, which command general confidence; but if the registration scheme should become law they will have to pay a yearly fee of half-a-crown for a certificate, which reduces good, bad, and indifferent nurses to a uniform dead level, and nobody will be able to say from any particular nurse's diploma whether she is the best nurse in the profession or the worst."

"AN IMPORTANT MEETING."—A short time ago the *Evening News and Post* had a little note anent registration, not too favourable in tone, and immediately they were swooped down on by the British Nursing Association, and invited to an important drawing-room meeting, to be taught by the crush and enthusiasm there witnessed what was the general opinion of registration. The representative of the paper in question meekly attended the meeting, and this is how he describes it: "Sir J. Crichton Browne was to have presided and deliver an address. Drawing-room meetings are peculiar functions, however. Usually they are crowded with fashionable and philanthropic ladies. Very long speeches are made to an audience still as statues, and the solemnity of the gathering is awe-inspiring. But yesterday's meeting was novel. The eminent gentleman who was to have taken the chair could not come. Not that there was anything strange in that. But neither did the ladies who were to have filled the handsome drawing-room present themselves. It is not, perhaps, easy to gather together on a fine summer afternoon, in the height of the London season, a goodly company of wealthy folk to listen to speeches upon subjects that have no special claim upon their attention. However that may be, yesterday's meeting consisted of about a dozen persons, and it resolved itself into what may be called a round-table conference. It was no meeting at all in the strict sense of the word; it was rather an informal conversation, guided by Dr. Bedford Fenwick, who performed Sir J. Crichton Browne's presidential duties, and led by Miss Wood." Surely the last sentence must rouse a smile on the face of anyone who knows the inner workings of the B.N.A.!

THE NEW HOSPITAL FOR WOMEN.—Mrs. Garrett Anderson is indefatigable in her efforts in aid of this hospital. She lately spoke at a drawing-room meeting at Mrs. Cartwright's at Richmond, and also at a meeting at St. Leonards. After Mrs. Anderson's lecture, Rukhmahai gave a short address. This lady spoke English with considerable ease and purity, and she urged on her hearers the enormous importance to the health and life of her sisters in India of their being supplied from England with suitable medical help. Owing to the customs and prejudices of her native land, such help can only come from women, and she entirely supported the lecturer's opinion—that there is in India an increasing demand for women doctors, and that the new hospital in the Euston-road will prove a most efficient way of providing the much-needed supply of a thoroughly trained and experienced medical staff.

WOMEN AS GUARDIANS.—After the account of imbeciles as nurses, it is not strange we should take an interest in lady guardians. The annual general meeting of the Society for Piomoting the Return of Women as Poor Law Guardians took place on June 27, at the Westminster Palace Hotel, Mr. J. G. Talbot, M.P., in the chair. In their report the committee expressed the satisfaction they felt at the considerable increase that had taken place in the number of women guardians. The Strand had returned three, Clapham and Battersea three, Hammersmith one, Hampstead one, and Greenwich two. Successes had been attained in other unions, and altogether there were twenty-eight women guardians in London. The most encouraging successes, however, had been achieved in the country. Southport had returned four, Manchester three, Hastings two, Richmond two, Leicester one, Huddersfield two, and the East Preston Union, in Sussex, one. There were in all forty-three women guardians in the country and five in Scotland, making seventy-six throughout the United Kingdom.

IMBECILES.—Not as patients, dear readers, but as nurses! Surely the folly of man can no further go than to appoint as nurses those who themselves require nursing and care. Unless we had seen it in black and white we could never have believed such folly could exist in any part of England; but here, in the report of the last meeting of the Atcham Board of Guardians, the chairman openly states it has been the constant practice of the institution to appoint imbeciles as assistant nurses. After such a confession, it is only natural to read of the recent suspicious death of an inmate, and that there is not sufficient evidence to show how the injuries from which that inmate died were caused. So far as we can gather, there is only one paid nurse to the infirmary, and it is her duty to visit each ward four times a day. Even visiting a ward four times a day cannot make of imbeciles good probationers, nor can it cause much comfort to the poor patients who perhaps require skilled attention. It also makes the matter worse (if possible) to know that the doctor had discharged the imbecile attendant who waited on the inmate who died, as unsatisfactory, but that she had been sent back to act as "nurse" by the master. Surely the people of Atcham will shortly arise from their sloth and elect some sensible women on to the Board of Guardians, who will see that poor old paralytic inmates of the infirmary cannot have their bones broken without someone being brought to justice.

### Notes from Australia.

*(By Our Own Correspondent.)*

Sydney, May 15.

This time I am writing to you from Sydney, a city of gardens and noble buildings. Hospitals are numerous, and are built of a soft yellow stone, which adds to their beauty. Prince Alfred Hospital is the finest in the colonies, both for size, situation, and modern appliances. The Sydney Hospital is older, but at present looks uncomfortable, with an unfinished stone front, and a wooden back, which is both hot and inflammable. Sir Henry Parkes, the Premier, visited it on April 29, and inspected all the wards. The Premier was informed by the architect that, so far, no damage had been suffered by the foundations put in several years ago, there being nothing more serious than a mere discolouration in places. If the buildings that had been begun were completed, 200 beds could be made up, and if the whole of the designs were carried out, 264 patients could be accommodated. About £69,000 had already been spent on the new buildings, and in providing accommodation for patients during the time those works were in hand. To finish in stone, the structures which had been started would cost £80,000; in brick £60,000; that would give a building of four floors. Sir Henry Parkes, before leaving, promised to bring the matter before the Cabinet, and on May 2 the directors handed in the following scheme: An emergency hospital with from 100 to 150 beds, a general hospital with from 350 to 420 beds, a lock hospital with from 20 to 30 beds, a hospital for chronic and incurable cases, a hospital home for consumptives, and a convalescence hospital. They contended that special provision should also be made for fevers and infectious diseases. These suggestions included much accommodation which already existed or was being brought into existence, but the directors urged that it was necessary that public provision should be made for the following objects: 1. An emergency hospital. 2. The addition of two or three additional pavilions to the general Prince Alfred Hospital to accommodate typhoid and additional general cases. 3. A hospital for chronic and incurable diseases. 4. A lock hospital for women. 5. A home for consumptive patients. When this scheme is carried out there will be some good posts as matron going.

The present Children's Hospital at Glebe Point is really two ordinary houses thrown into one, and it is intended to shortly build a new hospital at Randwick, where there would be a good view and delicious sea air. I can echo all the good reports of Miss Lobb, the matron, who is greatly beloved, as she deserves to be.

There is a "Home and Training School for Nurses for the Sick," 140, Phillip-street. It is doing good but up-hill work. In Sydney, as in England, scientific nursing has to win its way by much patient perseverance; there is a want of appreciation and understanding of a nurse's true work that is very trying to individuals.

One of the most interesting events of the past month was the laying of the foundation-stone of the Carrington Centennial Hospital for Convalescents, at Camden, near Sydney. The ceremony was performed by Lord Carrington in the presence of a large number of visitors, most of whom had arrived by special train from Sydney. The hospital originated in Mr. W. H. Paling's munificent gift of a large estate, accompanied by a present of £10,000, with which to start the building itself. The Freemasons of New South Wales, fired by a spirit of emulation, decided on erecting a cottage hospital on a portion of the property, the whole of the expense being borne by the members of the mystic craft, and the first stone of this building was laid by Lord Carrington immediately after laying the foundation-stone of the Centennial Hospital. The central block of the proposed group of hospital buildings, when complete, will accommodate 89 patients (49 males and 40 females), provision being made for a future extension to accommodate an additional 24 patients. The plan has been worked out with a view to the greatest simplicity of supervision and economy of management. The visitors' and the matrons' rooms will occupy the right and left portions under the central tower, the doctors' room and dispensary being also centrally placed, and close to the entrance. Care has been taken in separating the sexes, a different entrance being provided for each at the opposite ends of the building.

Turning to Melbourne once more, the following are the new rules with regard to nurses, submitted to the Committee of the Alfred Hospital:

1. That the period for nurses under training be extended from one to two years.
2. That for the first six months the pupil nurses be termed "probationers," for the second "pupils," and after the first year "nurses."
3. That the various grades be distinguished when on duty by a distinctive badge or dress.
4. That during the term of training, each nurse shall perform night duty for at least six months, not necessarily consecutive.
5. That for the first six months of her probation the pupil shall not receive any payment; but if at the end of that term she passes a primary examination, she shall receive for the second six months pay at the rate of £20 a year; for the third six months, at the rate of £25 a year; and for the fourth six months, at the rate of £30 a year.
6. That the foregoing salaries be paid on the distinct understanding that no extra pay will be allowed for any special or night duty that the pupil may be required to perform.
7. That the pay of assistant nurses—other than pupils—shall commence at £35 a year, and increase at the rate of £2 10s. annually up to £45 a year.
8. That the pay of head nurses shall commence at £50 a year, and increase at the rate of £3 for two years, and £4 for the third, up to £60 a year.
9. That the foregoing rules, so far as they affect the nurses at present under training, shall not be retrospective, save with the consent of any of the said nurses, but that the increased rate of pay to the head and assistant nurses date from the adoption of this report.
10. In conclusion, the committee endorsed the recommendation made to the managers on 29th June, 1888, that an assistant matron should be appointed to help Mrs. Strong and preside over the nurses' table.

Dr. Maffey inaugurated another series of popular lectures on nursing subjects to a large audience of ladies, at the Assembly Hall, Collins-street, yesterday. The lecturer pointed out that rules for proper nursing in typhoid fever postulated thorough and efficient ventilation of the sick-room, the absence of all unnecessary furniture, the systematic use of proper disinfectants, and avoiding as far as possible prolonged contact with the patient. With regard to disinfectants, widespread ignorance existed, and deodorants and antiseptics were commonly but erroneously regarded as true disinfectants capable of killing the germs of the disease. The most useful agents for this purpose were fumes of sulphur, chloride of lime, corrosive sublimate, sulphate of copper, and carbolic acid, which should all be carefully removed from all possibility of being taken by the patient in mistake. At the end of a fever illness the room should be disinfected with sulphur fumes, and the paper stripped from the walls and burned. The course is to consist of six lectures, all on fever nursing, and within the comprehension of all, yet not so elementary as to be of little use. The tickets for the course cost 5s., and the proceeds go to the Girls' Friendly Society.

The meeting in support of the movement for an increase of the city hospital accommodation, in the Town Hall, on April 30, was unanimous and enthusiastic. The Acting-Governor (Sir William Robinson) said the fact that nearly 300 sufferers had been refused admission to the Melbourne Hospital during the present year was a serious blot upon the fair fame of the great metropolis of Australia. It was resolved that, to meet immediate wants, at least £10,000 is required, and every citizen was urged to contribute in proportion to his means. Donations to the amount of £1,800 were promised at the meeting, amongst them the following: His Excellency the Acting Governor, £50; Mr. James Mason (when the first stone is laid), £1,000; Mr. Hugh Peck, £100.

The general meeting of the Medical Students' Society was held lately at the Melbourne Hospital. There were sixty-four members present, and Prof. Allen, president of the society, occupied the chair. Reference was made to the steady advance which the interests of the Medical School had made during the past year. During the year the Alfred Hospital had taken active part in teaching, and demonstrations had been regularly given. Clinical lectures had been continuously delivered at the Melbourne Hospital, and several improvements had been introduced into the curriculum of that institution. The society numbers 142 members, and does much for the advancement of medical knowledge.

### Dare We Utter Our Opinions?

Hitherto freedom of speech has been the universal rule in England, but it seems that there is arising in the ranks of medical journalism a party who, while they themselves indulge in the utmost license in their criticism of all who differ from them, would use any means to keep others from uttering their convictions. We recently received a pamphlet by Miss Lückes, which by this time is doubtless in the hand of many of our readers. The title is "What Will Trained Nurses Gain by Joining the British Nurses' Association?" and the writer expresses very simply and clearly her opinions on the matter. It seems incredible that any journal should think it necessary to judge the pamphlet otherwise than on its merits. Yet this is what we find in the *Lancet* of June 15:

"We have been much surprised to receive a pamphlet, signed Eva C. E. Luckes, matron of the London Hospital, inveighing against any form of registration being initiated for nurses. We should not have deemed this pamphlet worthy of notice, except that it is apparently issued semi-officially. . . We therefore should be glad to know whether this pamphlet has been issued by authority or not. If not, as we earnestly hope, it should be made plain to the public that this is the case, and thus free the London Hospital from the false and altogether untenable position in which it is placed."

We do not think the position of the London Hospital is nearly so shaky as the grammar of the *Lancet*. And we are very sure that the authorities of the London Hospital will not at the *Lancet's* bidding show discourtesy to a lady who fills her post so admirably, and has certainly the right to speak with authority on any question affecting nursing and nurses.

But for virulence of hate the *Lancet* is to the *Nursing Record* "as moonlight unto sunlight and as water unto wine." This is what the self-styled "representative organ the nursing profession" says of the pamphlet:

"Had it been issued by Miss Luckes in a private capacity it would probably have attracted little or no notice or comment in the nursing world. But as it is thus issued officially it at once acquires a certain significance, and as the representative organ of nurses, we must accord it our careful consideration. Whether the House Committee of the London Hospital have acted wisely in authorising this extraordinary production, we may say, at the outset, appears to us to be extremely doubtful. We should not be at all surprised if it resulted in alienating from their great institution a large measure of pecuniary support, and in arousing a very powerful professional and public feeling against themselves individually."

This is taken from the editorial. In the paragraphs collected under the head of "Nursing Echoes"—echoes, we believe, never repeat the whole of any information confided to them, but only mutilated fragments—we find the following:

"One cannot believe that the committee of the London Hospital, who are incessantly proclaiming the poverty of that institution, can have authorised funds, given by the charitable to provide for the sick, to be expended in attacking this women's association. . . . . Mr. Editor tells me that he proposes to criticise the pamphlet, as it is evidently issued by the authority of the managers of the London Hospital."

It is, therefore, very far from clear what the *Nursing Record* does believe. But, assuming that "Mr. Editor" thinks that Miss Lückes's pamphlet is approved by the managers of the London Hospital, he has the audacity to threaten them with social ostracism, and with the loss of pecuniary support for the institution they are interested in. Of course, this attempt at terrorism is absurd. The House Committee of the London Hospital will not insult Miss Lückes to please the *Lancet*, nor will the public refuse help to an important and valuable hospital because "Mr. Editor" of the *Nursing Record* would fain divert the stream of charity into, let us say, the empty coffers of the British Nursing Association. But though the threat is futile and absurd, we feel bound to say how unjustifiable we consider it. For the intention is that a certain small clique of nurses shall control the whole profession, and put a gag in the mouths of all who differ from them. The British Nursing Association may vaunt all that it is going—some day—to do for nurses, and none shall forbid it. But if a conscientious woman dares to say what she honestly thinks, the authorities of the institution to which she belongs are asked to express their disapproval of her conduct.

It is needless for us to defend Miss Lückes. Those who know her know that she is not one who will be moved either by cajolery or coercion. The position she occupies was not won by favour, nor is it held by aught but the thorough and conscientious performance of her duty. And assuredly that position is not endangered by anything she has said, nor will her hold on the respect of the authorities of the London Hospital be shaken by the fulminations we have quoted. But we protest, in the name of the nursing profession, against any member of it, the highest or the lowest, being forbidden the free utterance of honestly-held opinions.

### Everybody's Opinion.

[*Correspondence on all subjects is invited, but we cannot in any way be responsible for the opinions expressed by our correspondents. No communications can be entertained if the name and address of the correspondent is not given, or unless one side of the paper only be written on.*]

### LADIES AS POOR-LAW GUARDIANS.

C. G. F. writes: The meeting of the Society for Promoting the Return of Ladies as Poor-Law Guardians was a very satisfactory one. Almost everywhere success has followed the work of the society. A meeting in Manchester, addressed by Miss Louisa Twining, resulted in three ladies being elected. Leicester for the first time has returned a lady, and other places are following the good example. Miss Lidgett's defeat at St. Pancras was largely due, she herself admitted, to the fact that she too readily believed the assurances of her friends that she would be placed at the head of the poll without a struggle. In Chelsea two ladies were rejected on account of their political opinions. This is very stupid. Even if it be true that

"Every boy and every gal
That's born into this mortal hive,
Is either a little Liberal
Or else a little Conservative,"

they generally have their political opinions knocked out of them by the hard blows of Fate before they reach the workhouse. Paupers can't do much to secure Mr. Gladstone's return to power, and I am sure I never heard of any of them joining the Primrose League. To reject a candidate, male or female, who will deal honestly with the ratepayer's money and promote the pauper's well-being, simply on account of politics, is an example of that confusion of functions which is a most unfortunate characteristic of the average mind. I suppose it arises from the habit of some politicians of expressing doubts of their opponents' sincerity, but it is a most mischievous notion. Mr. Edward Chambers, of Birmingham, told his sad experiences in the cause of lady guardians. Twice has he nominated ladies who have been elected, the result being each time that he found himself first in the list of defeated candidates. But he is ready to risk a similar mishap again. Miss Phœbe Blyth spoke of the advantages of the boarding-out system for pauper children, which is so much followed in Scotland, and with excellent results. Never more than three children are sent to the same house, oftener only one or two. They lead the hard-working open-air lives of peasants, they gain the feeling of self-dependence that is the best antidote to pauperism, and the result is that after many years' trial of the plan it is calculated that only 1 per cent. of these children ever come back to the workhouse. The meeting expressed great satisfaction at the support the society meets with from public opinion and the Press, but I confess that I think it dealt very shabbily with the Press last Thursday. The reporters' table was put *behind* the audience, and as most of the speakers were not practised orators, hardly a word reached it. The result was that all the male reporters left in a very short time; only two loyal ladies strained their ears and held on to the end. Surely this is both a discourtesy and a blunder. A discourtesy to invite representatives of the Press, and then put them where they cannot hear; a blunder to throw away the admirable gratuitous advertisement afforded by a good notice in the newspapers. The society cannot yet afford to do without that.

INFIRMARY NURSING.

RICHARD GRANT writes: I enclose you a notice of the late meeting of guardians, by which you will see that the excellent system of nursing in force at Atcham Union consists in giving the imbeciles charge over the sick and dying. There is one trained nurse lately appointed, but I doubt if she will ever stop where so much that is difficult, almost impossible, awaits her. There are 300 sick in the infirmary, the place is beautifully clean, if only the guardians could be brought to see that even paupers when on their death-bed should be skilfully nursed. How would these guardians like to face the mortal agony of death, tended by unskilled hands, watched over by an imbecile face?

CONVALESCENT HOME FOR HIP CASES.

MRS. S. MASTERMAN writes from Garston, Torquay: In answer to query 37 in last week's THE HOSPITAL, I write to say that I might be able to take the child with hip disease and abscesses for six months. I happen to have a vacancy from to-day in my "Nursery Hospital," Rose Hill, Babbacombe, Torquay. I opened the Rose Hill Nursery Hospital last September for children with chronic and incurable diseases, intending to have four cots only for Devonshire, Cornwall, and Somersetshire, but for months past have never had less than nine cots occupied. I prefer young children, but take them from three to ten years of age. Most of the work is volunteer work. I have an hon. lady superintendent and one lady nurse, also one trained nurse. The lady superintendent was trained at Great Ormond-street. I hope you will excuse this long letter, but I would like the "Rose Hill Nursery Hospital" to get known, for, although I have started it quite privately and never appealed to the public for money, if it is more known I may benefit a larger number of little sufferers, as there is no institution of the kind in the whole West and South of England. I have some "Name Cots," for which ladies pay £25 a year, and other children are admitted at 5s., 3s., and free according to circumstances. I enclose an envelope, and will be glad to hear all further details about the little boy in question.

### Children's Country Holidays Fund.

Answers should be sent to the Secretary of the Fund, 10, Buckingham-street, Strand, W.C.

### Wants.

(1) *Helpers at end of July wanted.*—London ladies or gentlemen who would give some time at the end of July, when most arrangements for sending the children away have to be made, will be warmly welcomed.

(2) *Help for Paddington.*—The council wishes to form a committee to deal with the poorer parts of Paddington, and would like the names of those who know the poor there.

(3) *Wanted Supervisors and Country Homes in Villages.*—The C.C.H.F. wants country residents who will find and supervise country homes from the middle of July till the middle of August; 5s. a week is paid for each child. The villages should be within 50 miles of London. Cottagers who will take the very poorest and make special arrangements to ensure cleanliness are especially needed.

### Vacancies.

The following vacancies are announced:

### Matrons.

Manchester Eye Hospital, £80.
Nottingham Children's Hospital, £60.
Newcastle Royal Infirmary, £100.
Southport Children's Sanatorium £50.

### Nurses.

Bedford Nurses' Institute, £26.
Birmingham Fever Hospital, £20.
Birmingham Eye Hospital.
Blackheath Nursing Institution.
Croydon General Hospital.
East Suffolk Hospital, Ipswich (two), £25.
Epsom Cottage Hospital.
Guildford Hospital, £20.
Harrogate Cottage Hospital.
Hollond Institution, Nice.
Hospital for Sick Children, Stepney, £25.
Holborn Union, £20.
Ipswich Hospital (two), £25.
Isle of Wight Infirmary, £22.
Jersey Nurses' Institute, £25.
Kingston Home, Hull.
North London Consumption Hospital, £23.
Northampton Institution.
Pendlebury Children's Hospital.
Shadwell Mothers' Lying-In Home, £30.
St. Leonards-on-Sea Cottage Hospital.
St. Saviour's Infirmary, £20.
Southampton Institute.
Stratford-on-Avon Hospital, £20.
Weston-super-Mare Institution.
Workhouse Infirmary Nursing Association.

### District Nurses.

Burton-on-Trent, £25.
District Home, Bolton, £25.
Hampton Wick, £65.
Manchester and Salford Nursing Institute, £25.
Whitchurch, Hants, £28.

### Probationers.

Bristol Royal Infirmary.
Central Ophthalmic Hospital.
Hospital for Sick Children, Stepney.
Truro Infirmary, £9.

### Death in our Ranks.

ON June 17th, at All Saints' Home, Margaret-street, London, W., of phthisis, Sister Georgina Frances, of All Saints' Community (Georgina Frances Emma), daughter of the late Lieut.-Col. Charles Somerset, C.B., formerly of the Cape Mounted Rifle Corps, late of the 78th Highlanders, and grand-daughter of the late Lieut.-Gen. Sir Henry Somerset, K.C.B., K.H.—21st June, entered into rest, at Trained Nurses' Institute, Nurse Jessie Paine, third daughter of John Paine, Horselydown, aged 29 years.

BRISTOL ROYAL INFIRMARY.—A special service was held in the Hospital Chapel on Sunday morning, June 23, which was attended by the Right Worshipful the Mayor (Sir Charles Wathen), the Mayoress, Aldermen Sir George Edwards, Mr. W. E. George, several town councillors, and many members of the Infirmary Committee and of the faculty. At the service the dedication of a brass memorial tablet to a nurse, lately deceased, took place. The chaplain (the Rev. Fairfax Goodall) officiated, and preached the sermon from St. Matthew xxv., 23, "Well done, thou good and faithful servant; enter thou into the joy of thy Lord." The tablet bears the following inscription: "In memoriam, Elizabeth (Bessie) Hunt, born 6th December, 1850, died 19th May, 1889, for 14 years the devoted charge nurse of No. 6 ward in this infirmary. Erected by members of the medical staff, nurses, and friends. 'It is well.'"

### Appointments.

[It is requested that successful candidates will send a copy of their applications and testimonials, with date of election, to THE EDITOR, The Lodge, Porchester-square, W.]

EAST LONDON DISTRICT NURSING SOCIETY.—Miss Brancker has been appointed matron of the new division of the E.L.N.S. This makes the fourth matron now working in East London, and each matron has some seven districts and nurses to superintend. Miss Brancker trained at St. Bartholomew's, and was appointed matron of Newark General Hospital in September last.

SANATORIUM FOR CONSUMPTION.—Miss Henrietta Mormet, late lady superintendent of Watford Cottage Hospital, has been appointed lady superintendent of the National Sanatorium for Consumption and Diseases of the Chest, at Bournemouth. Miss Mormet was trained at St. Bartholomew's.

THE BRECON INFIRMARY.—Miss Lucy White has been appointed matron of this institution. She was trained at Addenbrooke's Hospital, Cambridge, where she obtained her certificate in May, 1884. Since then she has been a nurse at the Sussex County Hospital, Brighton, for eighteen months; after then she was nearly a year at the Worthing Infirmary, being then appointed charge nurse to the Hastings and St. Leonards Hospital, where she has acted at *locum tenens* for the matron on more than one occasion. Miss White's testimonials are excellent, and we congratulate her on her appointment to the Brecon Infirmary, where her experience and talents are well calculated to secure the best interests of the institution and its patients.

### Notes and Queries.

TO CORRESPONDENTS.—1. Questions may be written on post-cards. 2. Advertisements in disguise are inadmissible. 3. In answering a query please quote the number. 4. If a private answer is desired, a stamped addressed envelope must be forwarded.

### Queries.

(40) *Cape Colony.*—To whom should I apply for a post at Cape Colony? *Anna.*

### Answers.

(37) *Provision for Hip Cases.*—In answer to "Kate," "M. F." begs to say cases of hip disease for boys under seven are taken at St. John's Home, Kemp Town, Brighton. For further particulars apply to Miss Borradaile, at the above address.

(37) *Convalescent Home.*—Apply to Miss Derham, Bushey Heath, Herts.—*V. M.*

(38) *Nursing in New York.*—Apply to the Mount Sinai Hospital, New York, or the Brooklyn Training School for Nurses.—*Yankee.*

(40) *Cape Colony.*—There is a hospital at Port Elizabeth, to which "Anna" might apply. There are many Edinburgh nurses there.

